# Blood donors' motivation and attitude to non-remunerated blood donation in Lithuania

**DOI:** 10.1186/1471-2458-6-166

**Published:** 2006-06-22

**Authors:** Ilona Buciuniene, Laimutë Stonienë, Aurelija Blazeviciene, Ruta Kazlauskaite, Vida Skudiene

**Affiliations:** 1 Master of Management studies department, ISM University of Management and Economics, Kaunas, Lithuania; 2 Department of Social Medicine, Kaunas University of Medicine, Kaunas, Lithuania; 3 National Blood Centre, Vilnius, Lithuania; 4 Department of Philosophy and social science, Kaunas University of Medicine, Kaunas, Lithuania; 5 Undergraduate studies department, ISM University of Management and Economics, Kaunas, Lithuania

## Abstract

**Background:**

In the Soviet period, the blood donation system operated in Lithuania exclusively on a remunerative basis. After joining the EU, Lithuania committed itself to meeting the EU requirements to provide all consumers within its boundaries with safe blood products made from voluntary unpaid blood donations. However, the introduction of a non-remunerated donation system may considerably affect donors' motivation and retention. Thus the aim of the current research was to determine blood donation motives among the present donors and investigate their attitude towards non-remunerated donation.

**Methods:**

A questionnaire survey of 400 blood donors. Survey data processed using SPSS statistical analysis package. Statistical data reliability checked using Fisher's exact test (p < 0.05).

**Results:**

Paid donors comprised 89.9%, while non-paid ones made 10.1% of the respondents. Research findings show that 93 per cent of the paid donors give blood on a regular basis; while among the non-remunerated donors the same figure amounted merely to 20.6 per cent. The idea of the remuneration necessity is supported by 78.3 per cent of the paid donors, while 64.7 per cent of the non-remunerated respondents believe that remuneration is not necessary. The absolute majority of the paid donors (92%) think they should be offered a monetary compensation for blood donation, while more than half of the non-remunerated donors (55.9) claim they would be content with a mere appreciation of the act. Provided no remuneration were offered, 28.44 per cent of the respondents would carry on doing it, 29.6 per cent would do it only in emergency, 29.6 per cent would donate blood merely for their family or friends, and 12.3 per cent would quit it completely.

**Conclusion:**

Most respondents admitted having donated blood for the following reasons: willingness to help the ill or monetary compensation. Majority would consent to free blood donation only in case of emergency or as a family replacement, which leads to a conclusion that provided monetary remuneration is completely terminated part of the currently active paid donors would withdraw from this activity, which might seriously affect the national supply of blood and its products.

## Background

Over a million blood units are collected from donors every year; nevertheless, many more millions still need to be collected to meet the global demand and ensure sufficient and timely provision of blood [1]. This shows that blood donation is a highly relevant issue worldwide that calls for the government commitment to it as well as research in this field.

Generally donors are classified into the following categories: voluntary, family replacement and remunerated [2]. Donors are often analysed in reference to the frequency of donation: new or first time and sporadic or regular donors [2]. The safest donors are found among people who donate their blood voluntarily once or twice a year, purely out of altruism, and are self-aware of their unsuitability to serve as blood donors where there might be a slightest risk of causing health damage for blood recipients [3-6]. The possibility of catching hepatitis, however, is much higher among paid donors [7,8]. This might be accounted for by the fact that the latter come from environments where hepatitis cases are more commonplace; on the other hand, a person in need for money is more likely to conceal his/her true state of health, and monetary remuneration, which is often offered as a donor motivating means, might be highly appealing for people who live in desperate straits [9].

Most developed countries have well functioning systems of non-remunerated donation. East European countries unfortunately have inherited paid donation systems, with donors being offered compensation for blood donation. A 2001 analysis of blood donation systems in 17 European countries revealed that only five of them had exclusively voluntary non-remunerated donors (Finland, Republic of Yugoslavia, Slovenia, Spain and the UK) [10]. The Czech Republic, Greece, Italy, Macedonia, Romania, Croatia reported to have voluntary non-remunerated donors, but all or part of them were offered some sort of incentive (one or more days off work); whereas in Albania, Bosnia-Herzegovina, Bulgaria and Croatia blood supplies only partly (13–50 per cent) originate from really non-remunerated donors [10].

As to the general organization of transfusion, nine of the above countries reported to have hospital-based systems, 7 had national programmes, while Spain had a 'mixed' system. Speaking of donor promotion, most countries aid to have voluntary associations (Red Cross/Red Crescent in most cases) that are in charge of this activity.

Though paid donors and their family members are more likely to carry diseases that are transmittable through blood, such as HIV, Hepatitis B and C, and syphilis, over 50 per cent of blood donors in East European countries still come from the above social groups. Thus World Health Organization is encouraging its member states to establish national blood collecting agencies that would operate on the basis of voluntary, non-remunerable blood donation [5]. On the other hand, though monetary compensation is rejected by WHO, EU and Red Cross, monetary compensation for blood donation is permitted in Germany [11]. The reasoning behind this is that the frequency of transfusion-transmitted infection markers in the German donor population is low and, taking into account very likely increased blood supply shortages, all measures that would allow to improve the supply, including monetary compensation, should be discussed without prejudice [11].

Based on literature review, it can be stated that both developed and developing countries have problems with the non-remunerated blood donation system [12,13]. What encourages an individual to donate blood? Answers to this question make it possible for blood collection agencies to determine which individuals are likely to be new donors and enable to make predictions of prospective donors. The factors that influence an individual's decision to give blood is a collection of an individual's specific observable characteristics such as socio-demographic factors and unobservable characteristics such as the degree of altruism [14,15]. In order to facilitate the process of transition to non-remunerated donation, it is essential to examine and understand donor behaviour. According to some research findings, blood donation correlates with donor gender, place of birth, occupation and knowledge about donation as well as such social attitudes as health-related, structural and social-economic incentives [17].

### The development of the blood donation system in Lithuania

After joining the EU in 2004, Lithuania committed itself to meeting the EU requirement to provide all consumers within the EU with safe blood products made from voluntary unpaid blood donations. In the Soviet period, however, Lithuania merely had a paid donation system. As to the October 29, 2002 announcement of the National Health Board, non-remunerated donation, which is believed to constitute the principal guarantee of blood product safety, was still not common in the country and lacking adequate promotion [18]. In 2002 non-remunerated donation made 5.8 per cent of the national blood donations; in 2003 the figure amounted to 9.4 per cent, and 11.6 per cent in 2004 [18]. Thus in this context, it is really critical to look into the consequences that will be brought about by the termination of the donor remuneration system.

In parallel to other East European countries, Lithuania has quite a lengthy history of remunerated donation. In 1955 the Health Care Minister of the USSR ratified the following benefits for non-remunerated donors: a donor shall be given leave of absence on the day he/she donates blood, paid an average wage for the day and entitled to a free meal as well as granted a day off following each blood donation [19]. Thus it can be stated that a non-remunerated Soviet donor does not fall under the definition of a modern non-remunerated donor.

In 1992, two years following the regaining of its independence, Lithuania ratified a number of amendments to the Labour Code as concerns blood donors. The above document stated that donors were to be granted a leave of absence on the day they gave blood; where blood was given on non-remunerative basis, the donor was to be granted two days off and paid compensation from the social insurance funds amounting to their average three day pay [20]. The compensation system of the kind tied the amount of the remuneration to the donors' pay and provided them a possibility to get income from blood donations. Consequently blood-collecting institutions were flooded with people who had relatively high income. In 1995, the Government passed another law that amended the Labour Code article on blood donation, according to which donors under social insurance were to be paid a remuneration amounting to a three day national pay average [21].

In 1996 the Law on Blood Donation was passed, according to which blood was to be donated on the basis of voluntarism, anonymity and non-remuneration; however, Article 7 stated that blood donors were to be paid a remuneration amounting to a two day average pay and a leave of absence on the day blood was donated [22]. In 1997 the amount of remuneration was reduced to a one-day average national pay [18].

It was in the amendments to the Law of Blood Donation as of October 9, 2003 that for the first time donors were granted a right to non-remunerated donation of blood or blood components [22]. The above law also ratified a new donor compensation order and committed the state to promote non-remunerated donation.

In 2004, the Lithuanian Government ratified new remuneration regulations, which stated that upon a donor's request the latter was to be paid 40 litas (11.6 euro) from the national budget funds to compensate his/her travel and supplementary nourishment expenses [18]. The above regulations hold good to the present day. Besides the same year Lithuanian Health Ministry ratified a non-remunerated donation program and initial funds were allocated for its organization [22].

### Blood donation: management and promotion

In 2004, there were 3 blood transfusion departments (in hospitals) and National Blood Centre consisting of 5 blood collection centres in Lithuania, each of them operating within a certain geographical area and providing services to residents within a respective area. The five centres perform the following activities: blood donor recruitment, blood collecting, and preparation of blood products. Two of the above five blood collecting centres belong to hospitals, thus quite a part of their donors are family replacements. The remaining three blood centres are independent blood collecting organizations, each having offices in a number of cities and towns within their area of operation.

Blood collection centres operate in all cities and regional centres, and anyone willing to donate blood may do it at a centre that is of greatest convenience for him during the centre's office hours. Each donated blood unit is tested against the markers of such transfusion-transmittable diseases as Hepatitis B and C, HIV and syphilis. Where positive results are obtained, donors are informed of the fact and removed from donors for good.

**Research objective **is to identify the motives that serve as blood donation drivers among present donors as well as their attitudes to non-remunerated donation, as the understanding of their motivation drivers is crucial for the improvement of donor recruitment and retention effectiveness.

## Methods

### Data collection

Kaunas University of Medicine Bioethics Centre granted the ethical approval for the study. Kaunas Blood Centre granted the approval for the data collection. Research data was collected through a questionnaire survey. The instrument was developed so as to identify the socio-demographic characteristics of donors, look into their self-assessment of their participation in the donation activities, predict the number of donors who might possibly withdraw from blood donation if not given any remuneration, and determine possible ways to gradually re-orient current donors from paid to non-remunerated donation and encourage new members of the public to enter the blood donation program.

### Sampling

The research was carried out in Kaunas Blood Centre, one of the three donation centres under the National Blood Centre. In 2004, donors of the above organization comprised 27.81 per cent of the overall body of the Lithuanian donors (respectively 11,050 persons out of 39,733). It is also noteworthy that the above centre performs blood-collecting activities in 10 Lithuanian cities that cover 34 per cent of the national population. The Center is managed by the Lithuanian and American joint stock capital company *UAB Binational Plazma*, the founders of which are the Lithuanian Ministry of Health and US corporation Trimar. The survey was conducted during routine blood collection in the period of five working days, September 2004, in the health care establishments of the ten above-mentioned Lithuanian cities. Respondents were asked to fill in a questionnaire after blood donation; completed questionnaires were put in a box in the blood collection centre's reception. The research sample comprised 380 respondents (95% confidence level). All in all, 400 questionnaires were distributes and 380 filled-in returned, out of which 334 were usable for research purposes (response rate 83.5 %; confidence interval 5.34).

The collected survey data was processed using the SPSS statistical analysis package. Statistical data reliability was checked using Fisher's exact test (p < 0.05).

## Results

The analysis of the research data demonstrated that paid donors (300 persons) by large dominate volunteers (34 individuals), respectively 89.9 and 10.1 %. These findings are parallel to the national statistics (88.4 and 11.4% in 2004) [18]. The majority of both groups included donors 18 to 30 years of age. As to the respondents' level of education, the larger part of paid donors had secondary education, whereas volunteers had higher education. Both paid and non-remunerated donors came mostly from urban areas. Gender wise, 76.5 per cent of the non-remunerated donors were females, whereas the greater part of paid donors consisted of males (54 %). As to the income level, the larger part of paid donors came from a low-income social group. A statistically significant difference was determined in the respondents' distribution based on their occupation: 82.3 per cent of non-remunerated donors were under employment, whereas those under employment among the paid donors comprised merely 57.3 per cent of the respondents. This leads to an assumption that donors who are either students, unemployed, or persons with the lowest income are mostly concerned about getting compensation in return to blood donation, which indicates that both one's occupation and income level bear significant impact on the donor's decision to donate blood in exchange for a monetary compensation.

As to the motivation drivers of the first time donors, these were found different among paid and non-remunerated respondents. The first group reported to have been mainly inspired to take this action by certain personal considerations such as willingness to earn some money, find out their blood test results, help family or relatives in need, or sheer interest of trying blood donation. The larger part of non-remunerated donors though was led to donation by public promotion means.

Statistically significant differences were determined in the distribution among paid and non-remunerated donors based on their blood donation frequency (see Table 2). Research findings show that 93 per cent of paid donors do it on a regular basis, while among the non-remunerated ones the same figure stands at only 20.6 per cent.

The question on the personal meaning ascribed to blood donation evoked such responses among the respondents as help for the ill (50 %), importance of monetary compensation (21.8 %), and noble duty (18.6%). Comparison of the responses among paid and non-remunerated donors revealed some statistically significant differences: half of the non-remunerated donors believe that being a donor helps the ill, and 46.9 per cent find it a noble duty. As to the paid donors, 53.3 per cent consider blood donation being help for the ill, 19.7 per cent do it to receive compensation, and only 16.7 per cent look upon it as a noble duty (see Table 2).

As to the respondents' attitudes to remuneration, 71.9 per cent of all respondents think that blood donation should be remunerable, 9.6 per cent consider it should not, while 18.5 per cent have no firm opinion. Further analysis revealed statistically significant differences in the attitudes of paid and non-remunerated donors' towards remuneration (see Table 2): 78.3 per cent of the paid ones think donors should be paid, while 64.7 of the non-remunerable ones say it should be done for free.

The attitudes of paid and non-remunerated donors to the varying forms of compensation also bear statistically significant differences (see Table 2). Paid donors find monetary compensation most appealing (92%), while non-remunerated donors consider an expression of thanks (55.9%) the most appropriate form of compensation.

Responses to the question on one's consent to donate blood without being given any form of compensation for it distributed as follows: 59.3 per cent of the respondents said they would do it only in emergency, 28.4 per cent would give it to anyone, while 12.3 per cent gave a negative answer. There were also some statistically significant differences in the distribution of the above responses between paid and non-remunerated donors (see Table 2): 79.4 per cent of the non-remunerated donors would donate their blood to anyone in need, while among the paid ones this figure amounts to merely 22.7 per cent.

Research also looked into the respondents' opinion on the effect of blood donation on one's health. 55.7 per cent of paid donors consider it a healthy activity for its revitalizing effect, while 52.9 per cent of the non-remunerated donors did not give it any thought at all (see Table 2).

## Discussion

According to some researchers [12,14,15], the main motivating factor that mobilizes prospective donors is their awareness of the patients' need for blood in combination to one's presumption that one day they may also find themselves in need of blood transfusion. Other research findings support the claim that altruism and awareness of the need are not strong enough motivation factors [23,24]. The present research shows that people donate their blood if they receive a call to do it, are informed of somebody's vital need for their blood, wish to test their health condition or get some earnings. Thus the above reasons should be taken into consideration when developing donor recruitment programs.

In 1995 Germany had a remunerable donation system, and it was feared that donors were recruited from among persons with a higher risk level of HIV and other diseases that are transmittable through blood. To determine the donors' preparedness to waive remuneration or accept a reduced one as well as other alternatives, research was carried out, the results of which were expected to assist in altering donation motives and withdrawing from remunerated donation [9]. The research results demonstrated however that 86.1 per cent of the respondents were against remuneration termination and 77.0 per cent would quit doing it under such conditions. Thus findings of the German research are highly congruent to those of the Lithuanian case presented in this paper, where 71.9 per cent of the respondents think that blood donation must be compensated, and only 28.4 per cent would consent to free blood donation to anyone in need of it, while 59,3 per cent of respondents would do it in case of emergency. The latest research carried out in Germany demonstrated that the system of remunerated donation cannot be terminated abruptly, as the risk of running short of blood supply is higher than the safety risk of remunerated donors [11].

What significance do donors attribute to blood donation? The greater part of Lithuanian donors sees this act as help for the ill (50%); a small part looks upon it as a honourable duty (20%). These results are parallel to research carried out in other countries [25,26], where repeat blood giving is approached mainly from the rational point of view, i.e. purposeful help for the ill, and only a small part of donors do it for emotional and personal reasons. However, it has to be taken into consideration that about one fifth of the Lithuanian respondents consider blood donation as a possible means of income.

It is also noteworthy that not all persons who have once donated their blood become repeat donors. Findings of earlier research show that 40 per cent do it as a one-time act [15]. In the Lithuanian case, the greater part of non-remunerated donors comprises persons who did it for the first time. Thus it is really crucial to focus donor recruitment strategies on the transformation of the first timers' into the repeat ones as well as the retention of the latter [27].

To promote non-remunerated donation, it is essential to build a positive image of the donor in the public and further develop donation as an act of charity. Thus good public relations is a crucial promotional means in blood donor recruitment and retention management. Community participation and involvement in blood donation could also be encouraged by paying public honour to the most active donors and charity events. Another possibility would be to employ mass media in providing information on blood donation and its positive effect on human health as well as the national supplies of blood and its components at national blood collecting centres.

Retention of donors is also largely dependent on donor satisfaction with blood collection services [2]. So it is vital to help them feel at home at blood centres. Another crucial aspect is making donors feel that their blood donations are useful for the community and appreciated by it.

It is also recommended to develop stronger cooperation with medical specialists who use blood or its components for patient treatment as well as GPs and Red Cross Society. Greater emphasis on the social benefits of blood donation may possibly lead to higher involvement of organizations and higher education establishments. Promotional activities of blood donation should also be adapted to rapidly evolving communication technologies.

Further research should be done into the attitude to the donation issue among non-donor population, which would enable to identify predominant prejudices or fears that contribute to the development of negative attitudes to blood donation. This information is relevant in the development of information packages for donor recruitment campaigns as well as the formation of a positive attitude towards blood donation [28,29].

## Conclusion

All respondents named help for the ill as the principal motivational factor. The second most significant driver makes monetary compensation among the paid donors and viewing donation as a noble duty among the non-remunerated donors. Repeat paid donors consider money and the applicability for the state pension the most appropriate remuneration form, besides monetary remuneration is of highest significance for students, unemployed and persons with low income.

Majority of paid donors would justify non-remunerated blood donation only where their family or friends were in need of it. Given remuneration were terminated, they would continue giving blood, but would do it less frequently. Thus it can be assumed that having completely terminated remunerable donation part of the presently active paid donors, who currently constitute 89.9 per cent of all donors, will withdraw from this activity. Under such circumstances, provision of blood and blood products in the country might be endangered.

As to non-remunerated donors, a greater part of which are under employment, majority of them believe that remuneration is not necessary.

## Competing interests

The authors(s) declare that they have no competing interests.

## Authors' contributions

IB is the primary investigator and senior scientific advisor on motivation and attitude issues. LS has been involved in the conception of the study, designed the study, contributed to data acquisition and assisted in the preparation of the manuscript. AB performed the theoretical survey, statistical analysis and interpretation of the data. RK assisted in theoretical survey, text and table revisions, and manuscript edition. VS has been involved in theoretical survey, table and text revisions, and supervision of manuscript composition. IB, LS, RK, VS assisted in providing a critical appraisal and review of the manuscript. All authors read and approved the final manuscript.

## Pre-publication history

The pre-publication history for this paper can be accessed here:



## Figures and Tables

**Table 1:** Socio-demographic characteristics of the respondents

**Characteristics**	**Non-remunerated**	**Paid donor**	***p *value**
**Characteristics**	**Non-remunerated donor group N (%)**	**Paid donor group N (%)**	***p *value**
**Gender:**			
female	26 (76.5)	138 (46.0)	<0.05
male	8 (23.5)	162 (54.0)	>0.05

**Total**	*34 (100.0)*	*300 (100)*	

**Age group:**			
under 30	18 (52.9)	123 (41.0)	>0.05
over 30	16 (47.1)	177 (59.0)	>0.05

*Total*	*34 (100.0)*	*300 (100.0)*	

**Level of education:**			
incomplete secondary	5 (14.7)	54 (18.0)	>0.05
secondary	8 (23.5)	123 (41.0)	>0.05
higher	21 (61.8)	123 (41.0)	>0.05

*Total*	*34 (100.0)*	*300 (100.0)*	

**Place of residence:**			
urban	29 (85.3)	225 (75.0)	>0.05
rural	5 (14.7)	75 (25.0)	>0.05

*Total*	*34 (100.0)*	*300 (100.0)*	

**Occupation:**			
student & unemployed	6 (17.7)	128 (42.7)	>0.05
employed	28 (82.3)	172 (57.3)	<0.05

*Total*	*34 (100.0)*	*300 (100.0)*	

**Income:**			
under 500 litas	14 (41.2)	204 (68.0)	<0.05
over 500 litas	20 (58.8)	96 (32.0)	<0.05

*Total*	*34 (100.0)*	*300 (100.0)*	

**Table 2:** Donors' attitudes toward blood donation

**Characteristics**	**Non- remunerated donor group N (%)**	**Paid donor group N (%)**	***p *value**
**First time donation motives:**			
Public promotion	23 (67.6)	80 (26.7)	<0.05
Personal considerations:	11 (32.4)	220 (73.3)	<0.05
Interested in getting money*		*47 (15.7)*	
Interested in trying*		*47 (15.7)*	
Interested in getting a state pension*		*8 (2.7)*	
Interested in getting blood test result*		*44 (14.7)*	
Advised by the family doctor*		*17 (5.7)*	
Family or friends were in need*		*45 (15.0)*	
Other*		*12 (4.0)*	
(* Due to small sample, data for non-remunerated donors is not specified)			

*Total*	*34 (100.0)*	*300 (100.0)*	

**Distribution of paid and unpaid donors based on donation**			
**frequency**			
First time	27 (79.4)	21 (7.0)	<0.05
Frequent donors:	7 (20.6)	279 (93.0)	<0.05
Once in 2 months*		*264 (88.0)*	
Once a year*		*8 (2.7)*	
Less than once a year*		*7 (2.3)*	
(* Due to small sample, data for non-remunerated donors is not specified)			

*Total*	*34 (100.0)*	*300 (100.0)*	

**Personal meaning ascribed to blood donation:**			
Helping the ill	17 (50.0)	160 (53.3)	>0.05
Being a donor is a noble duty	16 (46.9)	50 (16.7)	<0.05
Other:		90 (30.0)	
Receive a monetary compensation*		*59 (19.7)*	
A possibility for a free blood test*		*31 (10.3)*	
(* Due to small sample, data for non-remunerated donors is not specified))			

*Total*	*34 (100.0)*	*300 (100.0)*	

**Donors' attitude to remuneration necessity:**			
Blood donations must be remunerated	5 (14.7)	235 (78.3)	<0.05
Remuneration is unnecessary	22 (64.7)	10 (3.3)	<0.05
No opinion	7 (20.6)	55 (18.3)	>0.05

*Total*	*34 (100.0)*	*300 (100.0)*	

**Paid and unpaid donors' attitude to remuneration forms:**			
Compensatory means	15 (44.1)	276 (92.0)	<0.05
Appreciation of donation act	19 (55.9)	24 (8.0)	<0.05

*Total*	*34 (100.0)*	*300 (100.0)*	

**Donors' attitude to non-remunerated blood donation:**			
Yes, anyone	27 (79.4)	68 (22.7)	<0.05
Yes, in case of emergency	7 (20.6)	191 (63.7)	<0.05
No	0 (0.0)	41 (13.6)	-

*Total*	*34 (100.0)*	*300 (100.0)*	

**Donors' attitude to donation's effect on the donor's health:**			
Positive effect (body revitalization)	10 (29.4)	167 (55.7)	>0.05
Zero or negative effect	6 (17.7)	60 (20.0)	>0.05
Never considered the effect	18 (52.9)	73 (24.3)	<0.05

*Total*	*34 (100.0)*	*300 (100.0)*	
